# The Effects of Regulatory Lipids on Intracellular Membrane Fusion Mediated by Dynamin-Like GTPases

**DOI:** 10.3389/fcell.2020.00518

**Published:** 2020-06-24

**Authors:** Yeojin Moon, Youngsoo Jun

**Affiliations:** School of Life Sciences and Cell Logistics Research Center, Gwangju Institute of Science and Technology, Gwangju, South Korea

**Keywords:** regulatory lipid, membrane fusion, GTPase, astlastin, mitofusin, endoplasmic reticulum, mitochondria

## Abstract

Membrane fusion mediates a number of fundamental biological processes such as intracellular membrane trafficking, fertilization, and viral infection. Biological membranes are composed of lipids and proteins; while lipids generally play a structural role, proteins mediate specific functions in the membrane. Likewise, although proteins are key players in the fusion of biological membranes, there is emerging evidence supporting a functional role of lipids in various membrane fusion events. Intracellular membrane fusion is mediated by two protein families: SNAREs and membrane-bound GTPases. SNARE proteins are involved in membrane fusion between transport vesicles and their target compartments, as well as in homotypic fusion between organelles of the same type. Membrane-bound GTPases mediate mitochondrial fusion and homotypic endoplasmic reticulum fusion. Certain membrane lipids, known as regulatory lipids, regulate these membrane fusion events by directly affecting the function of membrane-bound GTPases, instead of simply changing the biophysical and biochemical properties of lipid bilayers. In this review, we provide a summary of the current understanding of how regulatory lipids affect GTPase-mediated intracellular membrane fusion by focusing on the functions of regulatory lipids that directly affect fusogenic GTPases.

## Introduction

Membrane fusion is a vital step of a variety of fundamental processes in the cell and can be defined as a merger of two membrane-enclosed compartments into a single compartment. Membrane fusion is catalyzed by either a single protein or a series of proteins. Two types of fusogenic proteins are involved in most intracellular fusion events: SNAREs catalyze most of the membrane fusion events that occur during intracellular vesicle trafficking, while membrane-bound GTPases mediate the homotypic fusion of organelles such as the endoplasmic reticulum (ER) and mitochondria. These GTPases belong to a dynamin-like GTPase superfamily with conserved domain compositions and structures (Yan et al., 2015). The members of this family are mechanochemical GTPases that participate in the fusion and fission of membranes (Praefcke and McMahon, 2004). Here, we focus on dynamin-like fusogenic GTPases, including mitofusins (MFNs) and atlastins (ATLs), which share common features but act in different parts of the cell.

While proteins generally act as catalysts during membrane fusion, lipids have been long known to play a structural role. However, there is emerging evidence that lipids can also regulate membrane fusion events directly. These lipids, such as diacylglycerol, phosphatidic acid, phosphoinositides, and sterols, play more functional roles than structural roles during membrane fusion and thus are termed “regulatory lipids” (Fratti et al., 2004). The structures and physical properties of these regulatory lipids often differ from those of structural phospholipids; specifically, structural phospholipids take the form of cylinders with a typical phosphate head group and two acyl chains, while regulatory lipids display differential head group sizes and numbers of acyl chains and charges, resulting in different overall shapes of the lipids. In addition, regulatory lipids often contribute to the formation of microdomains on membranes, thereby affecting their physiochemical properties (Munro, 2003). These microdomains play an important role in membrane fusion by serving as fusion sites at which lipid rearrangement and bilayer mergers occur (Lang et al., 2008). Regulatory lipid-containing microdomains are believed to control membrane fusion mainly by changing the fluidity and curvature of the membrane, making it more prone to fusion (Zhukovsky et al., 2019). However, recent studies revealed that regulatory lipids also control membrane fusion by physically interacting with fusogenic proteins and thereby affecting their functions. There is indeed evidence for the direct involvement of regulatory lipids in GTPase-induced ER fusion and mitochondrial fusion through protein–lipid interactions. In this review, we describe current knowledge of the mechanisms by which certain regulatory lipids affect GTPase-induced intracellular membrane fusion.

## Mitofusin Is Involved in Mitochondrial Outer-Membrane Fusion

Mitochondria play a vital role in cellular homeostasis and survival by functioning as the key player in cellular ATP production, apoptosis regulation, and cell aging. Mitochondria normally exist as elongated tubules in the cytoplasm, undergoing constant fusion and fission (Bereiter-Hahn and Voth, 1994; Sesaki and Jensen, 1999; Shaw and Nunnari, 2002). Maintenance of the normal mitochondrial morphology is critical for their function, and mitochondrial dysfunction is associated with neurodegenerative disorders such as Parkinson’s and Huntington’s diseases (Chen and Chan, 2009). Because mitochondria are enclosed by outer- and inner-membranes with distinct roles, the mechanism by which fusion and fission of these two membranes are coordinated is a long-standing question. Fusion of the mitochondrial outer-membrane is controlled by the dynamin-like GTPases MFN1 and MFN2 in mammals and Fzo1p in yeast (Hermann et al., 1998; Rapaport et al., 1998; Ishihara et al., 2004; Koshiba et al., 2004), whereas OPA1/Mgm1p controls fusion of the inner-membrane (Alexander et al., 2000; Delettre et al., 2000; Olichon et al., 2003; Wong et al., 2003). Although fusion of the outer- and inner-membranes are mechanistically distinct events (Meeusen et al., 2004), they are tightly inter-regulated (Cipolat et al., 2004). In yeast, Fzo1p and Mgm1p cooperate to coordinate outer-membrane fusion and inner-membrane fusion (Sesaki et al., 2003; Sesaki and Jensen, 2004; Coonrod et al., 2007), and these two events are thought to be synchronized by Ugo1p (Hermann et al., 1998; Wong et al., 2003; Sesaki and Jensen, 2004). However, the exact mechanism involved in this process is still largely unknown, and a mammalian orthologue of Ugo1p is yet to be identified.

The first factor identified as a regulator of mitochondrial morphology was fuzzy onions (fzo) in *Drosophila* (Hales and Fuller, 1997). The mammalian homologues of fzo, MFN1 and MFN2, are similar in structure to each other, but these proteins seem to play separate roles in mitochondrial fusion (Santel and Fuller, 2001). Overexpression of MFN2 suppresses MFN1-induced mitochondrial tubulation (Eura et al., 2003). MFNs consist of a large N-terminal GTPase domain followed by two heptad repeat (HR) domains. Although it is generally accepted that the HR domains are separated by two transmembrane domains, thus both face the cytoplasm (Rojo et al., 2002; Li et al., 2019), a different topology of MFNs was also suggested (Mattie et al., 2018). In a working model for MFN1-induced fusion, MFN1 proteins in the fusing membranes form a homodimer via their GTPase domains upon GTP hydrolysis (Cao et al., 2017; Yan et al., 2018). This homodimerization induces a drastic conformational change of MFN1, resulting in close apposition and the subsequent merger of the membranes (Yan et al., 2018). The HR domains of MFNs (HR1 and HR2) are structurally similar to the SNARE domain of SNARE proteins, well-characterized fusogens involved in intracellular vesicle fusion (Bonifacino and Glick, 2004). Structural studies revealed that the HR domains of MFNs which consist of repeats of seven amino acids, form amphiphilic helices that potentially interact with each other by building coiled-coil structures, similar to the formation of trans-SNARE complexes between apposed membranes (Koshiba et al., 2004; Daste et al., 2018). Notably, HR1 and HR2 play distinct roles as follows: the HR2 domain forms an antiparallel dimer with another HR2 domain on the opposing membrane, which mediates docking between the two membranes (Koshiba et al., 2004), whereas the amphiphilic property of the HR1 domain enables it to bind to the surface of the membrane and perturb its structure, thereby facilitating membrane fusion (Daste et al., 2018). Although this working model by which MFN1 mediates mitochondrial membrane fusion has been widely accepted, the exact mechanism by which the HR domains facilitate fusion remains largely unclear.

## Phosphatidic Acid and Mitofusin-Mediated Fusion

Phosphatidic acid (PA) constitutes approximately 5% of the mitochondrial membrane. PA has a relatively small head group and thus becomes a cone-shaped lipid that spontaneously induces negative membrane curvature when present in lipid bilayers (Kooijman et al., 2005). There are two ways through which PA is incorporated into the mitochondrial membrane: first, the majority of PA molecules are transferred from the ER to the mitochondrial outer-membrane, presumably through ER-mitochondrial contact sites, such as ERMES in yeasts (Murley and Nunnari, 2016; Petrungaro and Kornmann, 2019); second, a smaller number of PA molecules are generated in the mitochondrial membrane directly through enzymatic conversion of cardiolipin (CL) by mitochondrial phospholipase D (MitoPLD) (Choi et al., 2006). PA influences both fusion and fission of the mitochondrial outer-membrane, although its exact roles in these processes remain poorly characterized (Choi et al., 2006; Adachi et al., 2016). One plausible role of PA in membrane fusion is the introduction of negative curvature into the membrane, making its shape more favorable for fusion (Frohman, 2015). MitoPLD also seems to be important for mitochondrial outer-membrane fusion as follows: overexpression of MitoPLD aggregates mitochondria, indicating fusion of these structures, and RNAi-mediated knockdown of MitoPLD dramatically decreases mitochondrial fusion (Choi et al., 2006).

**FIGURE 1:**
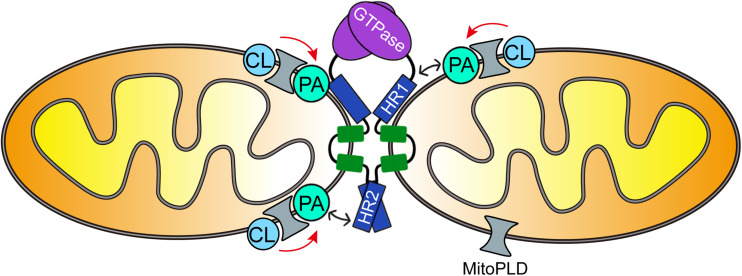
Schematic model of mitochondrial outer-membrane fusion. Mitochondrial phospholipase D (MitoPLD, gray) converts cardiolipin (CL) to phosphatidic acid (PA) on the mitochondrial outer-membrane, thereby increasing the concentration of PA at the site of fusion. PA then interacts with the HR domains of mitofusins (blue) and recruits them to the fusion site to facilitate membrane fusion.

Although there is no direct evidence that PA physically interacts with MFN1 to mediate membrane fusion, overexpression of phospholipase A1, which converts PA to lysophosphatidic acid, triggers mitochondrial fragmentation, while its suppression induces elongation of mitochondria (Baba et al., 2014), suggesting that mitochondrial fusion and fission depend on the level of PA in the mitochondrial outer-membrane. Notably, PA interacts directly with the N-terminal amphipathic helix of the SNARE Spo20p, a yeast homologue of mammalian SNAP25, recruiting it to the site of fusion (Nakanishi et al., 2004; Horchani et al., 2014). Since the HR domains of MFN also contain 2 conserved amphipathic helices and bind to the lipid bilayer, it is possible that they also associate with PA directly to facilitate mitochondrial outer-membrane fusion (Figure 1; Cohen and Tareste, 2018). A direct interaction between PA and Ugo1p, a protein involved in the coordination of mitochondrial inner- and outer-membrane fusion, has been reported in yeast, and PA is required for the biosynthesis of Ugo1p (Vogtle et al., 2015). Thus, it can be speculated that PA promotes the generation of Ugo1p, thereby enriching Ugo1p at the fusion site where the yeast MFN Fzo1p is also recruited. Taken together, these studies suggest that PA can regulate MFN-induced mitochondrial outer-membrane fusion, although the exact mode of action remains yet to be clarified.

## OPA1 Is Involved in Mitochondrial Inner-Membrane Fusion

OPA1 is a major regulator of mitochondrial inner-membrane fusion, and its genetic mutation is the main cause of optic atrophy (Alexander et al., 2000; Delettre et al., 2000). Deletion or mutation of the genes encoding OPA1 and its yeast orthologue Mgm1p results in abnormal mitochondrial morphology (Olichon et al., 2003; Wong et al., 2003). OPA1/Mgm1p belongs to the dynamin-like GTPase family and includes a GTPase domain in the middle section, a transmembrane domain at the N-terminus, and a membrane-binding domain, called a paddle domain, at the C-terminus (Faelber et al., 2019). Although encoded by a single gene, OPA1/Mgm1p exists in the following two forms: the long isoform L-OPA1/Mgm1p and the short isoform S-OPA1/Mgm1p. Short isoforms are produced by proteolytic cleavage (MacVicar and Langer, 2016) and lack the transmembrane domain, thereby existing as soluble proteins in the intermembrane space of mitochondria. Although both the short and long forms participate in inner-membrane fusion (Meeusen et al., 2006; DeVay et al., 2009; Zick et al., 2009), they seem to play distinct roles. The short form readily hydrolyzes GTP to initiate membrane tethering, and its drastic conformational change triggers membrane fusion (Zick et al., 2009; Faelber et al., 2019). By contrast, although the long form lacks GTPase activity, it associates with and activates the GTPase activity of the short form. Furthermore, the transmembrane domain of the long form is required for its precise targeting to the mitochondrial inner-membrane (DeVay et al., 2009). However, a recent study revealed that the long form of OPA1 is sufficient to drive liposome fusion in a GTP-dependent manner (Ban et al., 2017), indicating that it also plays a direct role in fusion. Thus, although both forms of OPA1/Mgm1 are required for mitochondrial inner-membrane fusion (DeVay et al., 2009; Ban et al., 2017; Ge et al., 2020), it is unclear how they cooperate to mediate this process.

## Cardiolipin and OPA1-Mediated Fusion

**FIGURE 2:**
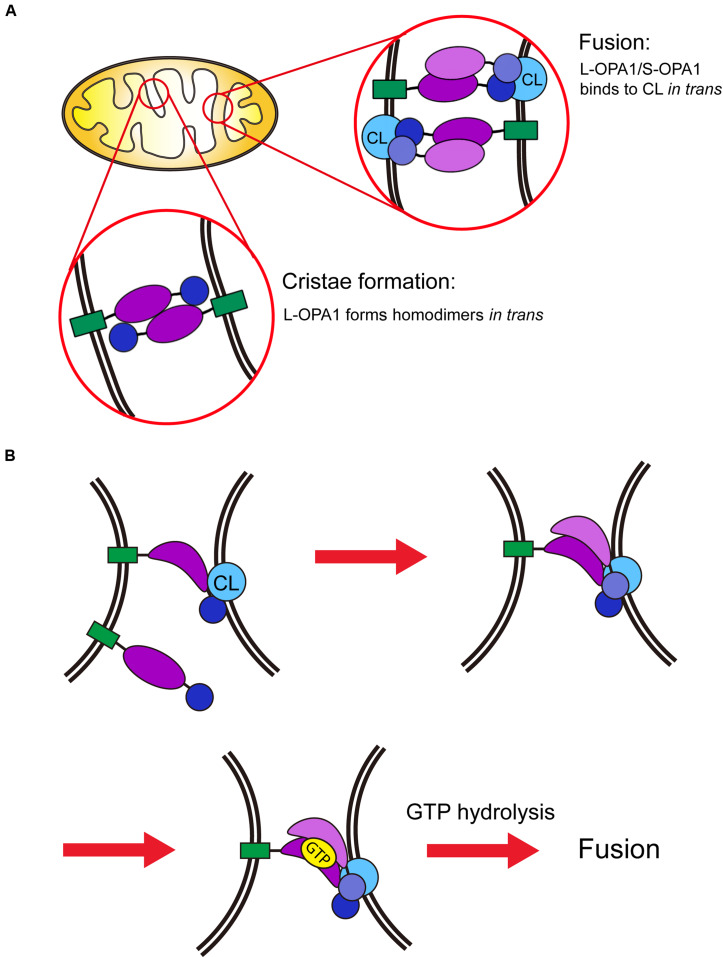
Schematic model of mitochondrial inner-membrane fusion. **(A)** GTP hydrolysis by OPA1/Mgm1p and the subsequent binding of OPA1/Mgm1p to CL are required for fusion of the mitochondrial inner-membrane. For cristae formation, the long form of OPA1/Mgm1p (L-OPA1/Mgm1p) forms a homodimer *in trans*. This process occurs independently of GTP hydrolysis. **(B)** L-OPA1/Mgm1p binds directly to CL *in trans*. This interaction induces the conformational change of L-OPA1/Mgm1p, allowing the short form of OPA1/Mgm1p (S-OPA1/Mgm1p) to associate with L-OPA1/Mgm1p. In turn, this interaction induces a conformational change of S-OPA1/Mgm1p to facilitate fusion.

Cardiolipin is an important lipid that comprises approximately 25% of the inner-membrane and approximately 4% of the outer-membrane phospholipids (Ardail et al., 1990; Horvath and Daum, 2013). Unlike other phospholipids, CL has a unique chemical structure; it contains two phosphate head groups and four acyl chains, forming a symmetric structure. A number of reports have emphasized the importance of CL in mitochondrial inner-membrane fusion. For example, the inactivation of enzymes involved in CL synthesis generally causes morphological defects of mitochondria (Matsumura et al., 2018). In addition, CL regulates the mitochondrial morphology directly by facilitating the assembly of the dynamin-like GTPase OPA1/Mgm1p (DeVay et al., 2009; Rujiviphat et al., 2009; Joshi et al., 2012; Ban et al., 2017). Moreover, CL stimulates the GTPase activity of S-Mgm1p in a concentration-dependent manner, as evidenced by the finding that GTP hydrolysis by S-Mgm1p was higher in liposomes containing 20% CL than in liposomes containing 6% CL (DeVay et al., 2009). Similarly, enhanced GTP hydrolysis and S-OPA1 oligomerization were observed in the presence of CL (Ban et al., 2010). Compared with the short form of OPA1/Mgm1p, little is known about the long form, mainly because L-OPA1 is difficult to purify for biochemical studies. However, in a recent study, recombinant L-OPA1 was successfully purified from silk worm, and its function was assessed *in vitro*. Strikingly, this study reported that L-OPA1 was sufficient to drive fusion of liposomes containing 25% CL in a GTP-dependent manner. This fusion requires heterotypic interactions between L-OPA1 and CL in trans; specifically, L-OPA1 in a liposome binds to CL in another liposome (Ban et al., 2017; Ge et al., 2020). This result may explain why fusion was observed between mitochondria from OPA1-depleted cells and those from wild-type cells (Ban et al., 2017, 2018). Thus, CL may serve as a binding site for S/L-OPA1 heterodimers, thereby enabling these proteins to tether membranes and induce the subsequent fusion (Figure 2A).

L-OPA1 induces fusion only when it interacts with CL on the opposite membrane in trans. Therefore, it has been suggested that the CL-binding region of L-OPA1 is required for its recruitment to CL-enriched microdomains to facilitate fusion (Ban et al., 2017; Ge et al., 2020). Furthermore, a recent structural study of S-Mgm1p revealed that the CL-binding site lies on the GTPase domain, and its positively charged residues on the surface participate in electrostatic interactions between negatively charged lipids (Yan et al., 2020). Therefore, it is possible that the interaction of L-Mgm1p/OPA1 with CL induces a conformational change in the protein to facilitate the formation of S/L-OPA1/Mgm1p heterodimers. It is also possible that this interaction enhances the GTPase activity of Mgm1p/OPA, which then supports efficient fusion (Figure 2B).

A similar mode of action in promoting yeast vacuole fusion was observed for the Phox homology domain of the SNARE Vam7p. This domain binds phosphatidylinositol 3-phosphate (PI(3)P) on the vacuolar membrane, resulting in the accumulation of Vam7p at PI(3)P-rich regions, where it forms trans-SNARE complexes with other SNARE proteins to promote vacuole fusion (Cheever et al., 2001). In addition, the interaction between PI(3)P and the Phox homology domain of Vam7p is thought to cause a conformational change in Vam7p, which may enhance its interaction with other fusion components (Cheever et al., 2001; Miner et al., 2016).

## Atlastin Is Involved in ER Fusion

The ER, a large but single organelle that spreads throughout the cytoplasm, is the major site of lipid synthesis, protein folding, and protein quality control (Baumann and Walz, 2001; Ellgaard and Helenius, 2003). Although enclosed by a single, continuous lipid bilayer, the ER exists in the following two distinct forms: a sheet like structure surrounding the nucleus and a tubular network dispersed throughout the cytoplasm (Voeltz et al., 2002). The tubular ER is a dynamic structure that constantly undergoes elongation, retraction, and fusion (Lee and Chen, 1988). The tubular structure of the ER seems to be important for its function because it enables distinct membrane contact sites with various organelles (Phillips and Voeltz, 2015). Maintenance of the proper morphology of the ER is thought to be important for normal cell physiology, and its disruption is often associated with neurological disorders such as hereditary spastic paraplegia (Namekawa et al., 2006; Salinas et al., 2008; Park et al., 2010).

Although the mechanism by which the tubular ER network is formed and maintained remains poorly understood, Yop1/DP1 and a class of proteins called reticulons are thought to play a critical role in generating the high membrane curvature required to form ER tubules (Voeltz et al., 2006; Hu et al., 2008). In addition, ATLs, which belong to the family of dynamin-like GTPases, are also thought to mediate the fusion of ER tubules (Orso et al., 2009) by forming three-way junctions of the tubules and thus generating the mesh-like structure of the ER. *Drosophila* ATL alone or yeast ATL (Sey1p) with either reticulon or DP1 is sufficient to recapitulate formation of the tubular ER network structure *in vitro* when reconstituted into synthetic liposomes (Powers et al., 2017). Furthermore, proteoliposomes reconstituted with purified *Drosophila* ATL, Sey1p, or the plant ATL Root Hair Defective 3 are able to fuse with each other, confirming that these proteins can function as genuine fusogens (Orso et al., 2009; Anwar et al., 2012; Zhang et al., 2013). However, human ATL1 is unable to induce liposome fusion, suggesting that additional proteins are required for ER membrane fusion in human cells (Wu et al., 2015). The fusogenic activities of the other human ATLs (ATL2 and ATL3) have not yet been investigated.

Atlastin family proteins contain a large N-terminal GTPase domain followed by three helical bundles, two transmembrane domains, and a short α-helix at the C-terminal end (Bian et al., 2011; Yan et al., 2015). The current model for ATL-induced membrane fusion is that upon GTP hydrolysis, the GTPase domain of ATL forms a homodimer with that of another ATL molecule on the apposed membrane, and their helix bundles then undergo dramatic conformational changes that bring the membranes into close proximity, which eventually induces the fusion of ER tubules (Bian et al., 2011; Yan et al., 2015; O’Donnell et al., 2017; Winsor et al., 2017). Although it is widely accepted that ATLs are sufficient to drive liposome fusion and are therefore the major fusogens for ER membrane fusion (Orso et al., 2009; Anwar et al., 2012; Zhang et al., 2013), a recent study using purified yeast ER microsomes suggested that additional factors are required for efficient ER fusion *in vivo*, at least in yeast (Lee et al., 2015). In this study, ER-resident SNAREs were critical for ER microsome fusion *in vitro* and for normal ER morphology *in vivo*. This finding is consistent with the observation that human ATL1 alone is insufficient to induce liposome fusion.

## Cholesterol and Atlastin-Mediated Fusion

Cholesterol has a small hydrophilic head group and a bulky steroid backbone, and is a vital component of biological membranes. Accumulating evidence supports the importance of cholesterol in various fusion events, such as exocytosis (Wasser et al., 2007; Linetti et al., 2010) and viral fusion (Klug et al., 2017; Lee et al., 2017). Cholesterol is thought to participate in membrane fusion mainly by altering the biophysical properties of the membrane, such as the fluidity, thickness, curvature, and stability of lipid bilayers (Yang et al., 2016). In addition, cholesterol may also regulate membrane fusion by interacting directly with fusogenic proteins. Consistent with this idea, cholesterol promotes clustering of SNARE proteins at the site of fusion (Murray and Tamm, 2011; Enrich et al., 2015). Furthermore, some SNARE proteins contain cholesterol-binding motifs, such as CRAC [**C**holesterol **R**ecognition/interaction **A**mino acid **C**onsensus sequence, (L/V)-X_1–5_-Y-X_1–5_-(K/R)] and CARC [an inverted CRAC motif, (K/R)-X_1–5_-(Y/F)-X_1–5_-(L/V)], in or near their transmembrane regions (Enrich et al., 2015), suggesting that cholesterol affects the function of SNAREs to facilitate membrane fusion by binding to them directly.

**FIGURE 3:**
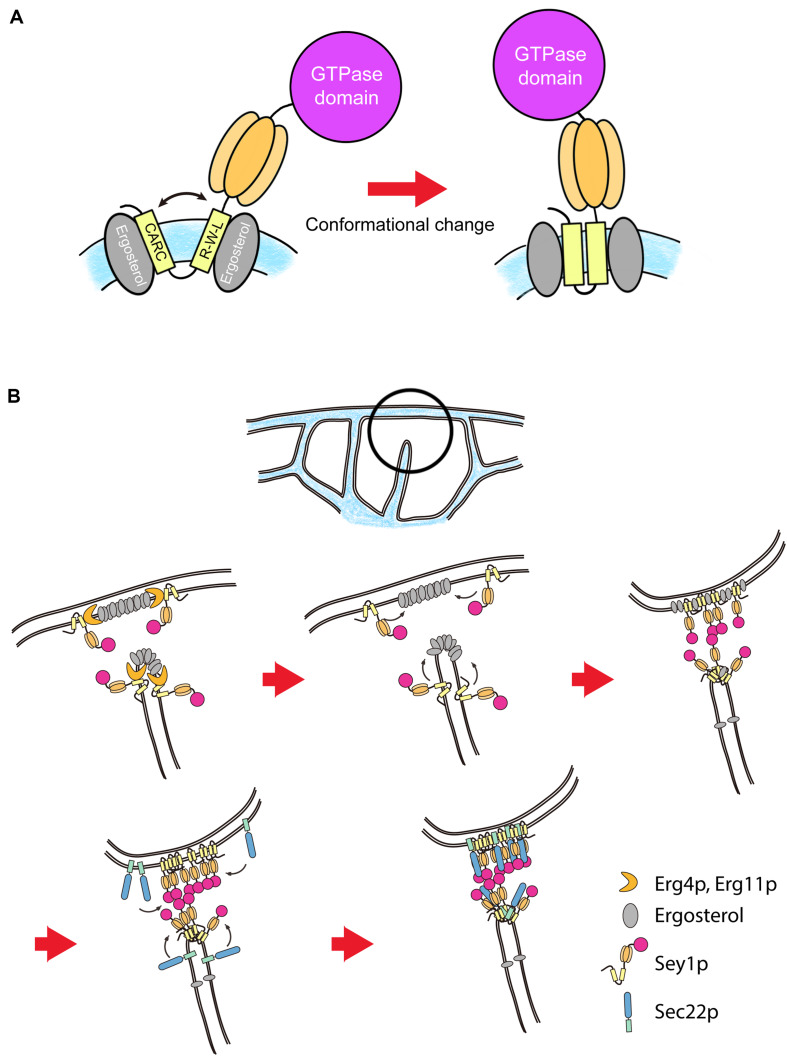
Schematic model of the role of ergosterol in Sey1p-mediated ER membrane fusion in yeast. **(A)** Sey1p interacts directly with ergosterol through its sterol-binding motifs, CARC and R-W-L. This interaction may promote transition of the transmembrane domains of Sey1p from an open, scissor-like configuration to a closed, parallel configuration. The conformational change may also increase the binding affinity of Sey1p for the ER SNARE Sec22p, recruiting more Sec22p proteins to the fusion site to enable efficient fusion. **(B)** Ergosterol is synthesized by a series of sterol biosynthetic enzymes, including Erg4p and Erg11p. Sey1p interacts with Erg4p/Erg11p and thus recruits them to the fusion site, increasing the local concentration of ergosterol. In turn, this process recruits more Sey1p and Sec22p proteins to the site of fusion, and this positive loop may greatly facilitate ER fusion.

We recently revealed that ergosterol (yeast cholesterol) affects ER membrane fusion by interacting directly with Sey1p (Lee et al., 2019). The transmembrane domains of Sey1p contain two sterol-binding motifs, the R-W-L motif (a combination of basic [R], aromatic [W], and aliphatic [L/V] residues) and the CARC motif (Figure 3A). Furthermore, disruption of these sterol-binding motifs abolished the binding of sterols to Sey1p, severely reduced ER microsome fusion *in vitro*, and disrupted the normal ER morphology *in vivo*. Although the exact mechanism by which sterols stimulate Sey1p-medited ER fusion remains unclear, one possibility is that the interaction between the transmembrane domain of ATLs and cholesterol (or ergosterol in yeast) causes conformational changes of ATLs, making them more favorable for fusion. Consistent with this idea, mutant Sey1p lacking the sterol-binding motifs is unable to interact with Sec22p (Lee et al., 2019), an ER SNARE involved in Sey1p-dependent ER fusion (Lee et al., 2015), supporting the notion that the binding of cholesterol to Sey1p affects the overall conformation of the protein, resulting in modification of its fusogenic activity as well as of the profiles of its interacting proteins (Figure 3A). Notably, the transmembrane domain of the SNARE synaptobrevin-2 exists as two distinct forms, an open scissor form and a closed, parallel form, depending on the presence of cholesterol. This conformational transition modifies the fusogenic activity of the protein by changing the curvature of the surrounding membrane and possibly promotes complex formation with other SNAREs (Tong et al., 2009). Because ATLs contain two transmembrane domains, it is plausible that their conformations are affected by the presence of cholesterol similarly to that of the transmembrane domain of synatobrevin-2. Furthermore, because potential sterol-binding motifs are found in all human ATL proteins, regulation of ATL activity by direct binding of cholesterol is likely to be evolutionarily conserved.

We also found that Sey1p interacts physically with Erg4p and Erg11p, enzymes involved in the biosynthesis of ergosterol, which raises the possibility that Sey1p acts to increase the local concentration of ergosterol at the fusion site (Lee et al., 2019). In turn, this process not only stimulates the pre-existing Sey1p molecules for efficient fusion, but also recruits more Sey1p molecules and interacting proteins such as Sec22p to the site of fusion (Figure 3B). In support of this concept, ER subdomains containing Rab10, which reportedly mediates fusion between ER tubules in mammalian cells, are enriched in ER enzymes that regulate phospholipid synthesis, including phosphatidylinositol synthase and choline/ethanolamine phosphotransferase 1, which converts diacylglycerol precursors to phosphatidylethanolamine and phosphatidyl-choline (English and Voeltz, 2013).

In addition to the direct participation of cholesterol in ATL-mediated ER fusion, structural and biochemical studies of *Drosophila* ATL have suggested that a direct interaction of the C-terminal tail of ATL with lipid bilayers plays an important role in ER membrane fusion (Moss et al., 2011; Liu et al., 2012). In one of these studies, deletion of the short C-terminal tail of *Drosophila* ATL almost completely abolished the fusion of phosphatidylcholine:phosphatidyl-serine (PC:PS) proteoliposomes (Moss et al., 2011). The C-terminal tail of ATL is predicted to form an amphiphilic helix, which is very likely to be embedded into the lipid bilayer, thereby affecting the curvature and the stability of the membrane (Drin and Antonny, 2010). Indeed, the hydrophobic residues of the C-terminal tail of ATL interact directly with the hydrophobic side of the lipid bilayer (Liu et al., 2012). Similar observations were made for the plant ATL Root Hair Defective 3, which contains a conserved C-terminal tail that is required for ER targeting and efficient ER membrane fusion, implying that the C-terminal region is inserted into the lipid bilayer, as seen in *Drosophila* ATL-mediated fusion (Sun and Zheng, 2018). Although it is unclear how the C-terminal tail of ATL functions during ER membrane fusion, its insertion into the membrane may perturb the lipid bilayer, making it more prone to membrane fusion (Liu et al., 2012; Faust et al., 2015). However, it was reported that the necessity of the C-terminal tail of ATL for membrane fusion became less stringent when phosphatidylethanolamine (PE), a non-bilayer-prone lipid, was added to PC:PS proteoliposomes (Faust et al., 2015). This result suggests that although the C-terminal tail of ATL facilitates fusion, it is not essential for ER membrane fusion *in vivo*, as ER membranes contain significant amounts of non-bilayer-prone lipids such as phosphatidylethanolamine, cholesterol, and diacylglycerol (van Meer et al., 2008). In particular, Sey1p-mediated liposome fusion is highly susceptible to the omission of PE or ergosterol (Sugiura and Mima, 2016; Lee et al., 2019).

## Discussion

This review describes the role of regulatory lipids in GTPase-mediated intracellular membrane fusion, focusing on examples of how these lipids affect proteins involved in membrane fusion processes. Some regulatory lipids facilitate membrane fusion by serving as an anchoring site for partner proteins and thus concentrating them at the site of membrane fusion, while others may bind directly to fusion proteins and modulate their fusogenic activity. Although lipids and proteins are both key players of membrane fusion, we have only just started to understand how their interactions control membrane fusion, and much remains to be clarified. A number of fusogenic proteins have potential lipid-binding domains or motifs; however, further studies are required to determine whether they indeed bind to lipids and how their interactions affect membrane fusion. In a recent report (Lee et al., 2019), we demonstrated that the yeast ATL Sey1p contains two sterol-binding motifs near its transmembrane domains. Disruption of these motifs severely abrogates Sey1p-mediated ER fusion, suggesting that the binding of sterols affects the fusogenic function of Sey1p. We also found that all three human ATL proteins contain two potential sterol-binding motifs. It would be interesting to investigate whether human ATLs associate directly with cholesterols, and whether this interaction influences their fusogenic activity. A study by Joji Mima’s laboratory showed that Sey1p-mediated liposome fusion is stimulated by other regulatory lipids, such as phosphatidylinositol and PA (Sugiura and Mima, 2016). It would therefore also be interesting to investigate how these lipids regulate Sey1p-mediated fusion. Compared with current knowledge of the role of regulatory lipids in ATL-mediated ER fusion, much less is known about how regulatory lipids control GTPase-mediated mitochondrial fusion. Recent advances in research tools for lipid studies and microscopy will guarantee a deeper and more comprehensive understanding of how regulatory lipids dictate GTPase-mediated intracellular membrane fusion events.

## Author Contributions

Both authors listed have made a substantial, direct and intellectual contribution to the work, and approved it for publication.

## Conflict of Interest

The authors declare that the research was conducted in the absence of any commercial or financial relationships that could be construed as a potential conflict of interest.
